# Identifying implementation strategies to address barriers of implementing a school-located influenza vaccination program in Beijing

**DOI:** 10.1186/s43058-023-00501-8

**Published:** 2023-10-11

**Authors:** Ruijie Yan, Xuejun Yin, Yiluan Hu, Huan Wang, Chris Sun, Enying Gong, Xin Xin, Juan Zhang

**Affiliations:** 1 School of Population Medicine and Public Health, Chinese Academy of Medical Sciences (CAMS) and Peking Union Medical College (PUMC), Beijing, 100730 China; 2 The George Institute for Global Health, University of New South Wales, Sydney, NSW 2052 Australia; 3 Faculty of Psychology, Beijing Normal University, Beijing, 100875 China; 4 Research Unit of Population Health, Faculty of Medicine, University of Oulu, 5000 Oulu, Finland

**Keywords:** School-located influenza vaccination, Consolidated Framework for Implementation Research, Expert Recommendations for Implementing Change, China

## Abstract

**Background:**

The school-located influenza vaccinations (SLIV) can increase influenza vaccination and reduce influenza infections among school-aged children. However, the vaccination rate has remained low and varied widely among schools in Beijing, China. This study aimed to ascertain barriers and facilitators of implementing SLIV and to identify implementation strategies for SLIV quality improvement programs in this context.

**Methods:**

Semi-structured interviews were conducted with diverse stakeholders (i.e., representatives of both the Department of Health and the Department of Education, school physicians, class headteachers, and parents) involved in SLIV implementation. Participants were identified by purposive and snowball sampling. The Consolidated Framework for Implementation Research was adopted to facilitate data collection and analysis. Themes and subthemes regarding barriers and facilitators were generated using deductive and inductive approaches. Based on the Consolidated Framework for Implementation Research—Expert Recommendations for Implementing Change (CFIR-ERIC) matching tool, practical implementation strategies were proposed to address the identified barriers of SLIV delivery.

**Results:**

Twenty-four participants were interviewed. Facilitators included easy access to SLIV, clear responsibilities and close collaboration among government sectors, top-down authority, integrating SLIV into the routine of schools, and priority given to SLIV. The main barriers were parents’ misconception, inefficient coordination for vaccine supply and vaccination dates, the lack of planning, and inadequate access to knowledge and information about the SLIV. CFIR-ERIC Matching tool suggested implementation strategies at the system (i.e., developing an implementation blueprint, and promoting network weaving), school (i.e., training and educating school implementers), and consumer (i.e., engaging students and parents) levels to improve SLIV implementation.

**Conclusions:**

There were substantial barriers to the delivery of the SLIV program. Theory-driven implementation strategies developed in this pre-implementation study should be considered to address those identified determinants for successful SLIV implementation.

**Supplementary Information:**

The online version contains supplementary material available at 10.1186/s43058-023-00501-8.

Contributions to the literature

- This study highlights the importance of the work in the pre-implementation phase. Theory-driven implementation strategies should be selected and applied based on the specific multilevel determinants to enhance the adoption, implementation, and sustainment of the school-located influenza vaccination (SLIV) program.
- Using the Consolidated Framework for Implementation Research (CFIR), this study highlights how the CFIR domains and constructs might play a positive or negative role, identifying barriers and facilitators to the implementation of the SLIV program in Beijing, China.
- Implementation strategies developed by applying the Expert Recommendations for Implementing Change (ERIC) will inform intervention development in an upcoming hybrid trial.

## Background

Influenza epidemics can cause three to five million severe cases and 290,000 to 650,000 respiratory disease-related deaths worldwide each year [1]. Young children are more susceptible to influenza, and it can quickly spread during influenza season in schools [2, 3]. A study in Beijing suggested that influenza incidence in children aged 5–14 was as high as 22% during the 2017–2018 influenza season [4].

The Global Influenza Strategy 2019–2030 highlighted seasonal influenza vaccination as the most cost-effective intervention for preventing infection and potentially reducing clinical severity [5]. There is a wealth of evidence showing that school-located influenza vaccination (SLIV) can increase the influenza vaccination rate among school-aged children [6–9], as well as decrease the incidence of influenza-like illness [9, 10], influenza outbreaks [11, 12], and student absenteeism [13, 14] in schools. Notably, during COVID-19, SLIV would be critical to reducing the unnecessary medical visits and hospitalizations of respiratory illnesses attributed to influenza among children, thus reducing burdens on the health care system.

The Beijing government has implemented school-located influenza vaccination program to school-aged children since 2007. Primary and secondary school students in Beijing are encouraged to receive annual influenza vaccinations [15], but the vaccination rate has remained lower than the average in developed countries. For example, in the influenza season of 2017–2018, the vaccination rate of children aged 6–17 in the USA was 58%, while only 47% in Beijing [4]. Additionally, the vaccination rate varied across schools in Beijing. For example, the influenza vaccination coverage among primary schools ranged from 31 to 77%, with over half of schools having less than 50% of students vaccinated during the 2019–2020 season. Notably, herd immunity for unvaccinated children and the wide population may occur when influenza vaccination coverage in school approaches 50% [4, 12, 16, 17].

Low coverage of influenza vaccination via SLIV is influenced by factors at the individual level and more broadly through contextual determinants including policies and organizational factors (e.g., schools) [18]. To maximize effective implementation, strategies should be selected based on identified multilevel determinants of implementation—i.e., the underlying factors that either facilitate or impede implementation. We conducted a qualitative study to explore the barriers and facilitators of SLIV implementation and to identify the potential SLIV implementation strategies based on implementation theory. The results of this study will inform the design of implementation strategies for enhancing SLIV delivery and improving influenza vaccination coverage in Beijing, which will then be tested in a hybrid type 2 cluster randomized controlled trial (Chinese Clinical Trial Registry, trial register no.: ChiCTR2200062449).

## Methods

### Design

The study was undertaken in two parts: part I—interviews of stakeholders to identify the barriers and facilitators of SLIV implementation in schools; part II—using the Consolidated Framework for Implementation Research (CFIR)—Expert Recommendations for Implementing Change (ERIC) matching tool to suggest possible strategies to mitigate the barriers and leverage facilitators that were identified in part I. Our research team consisted of researchers in epidemiology, respiratory infectious disease, qualitative methodology, psychology, and master’s students. The study was approved by the Institutional Ethics Committee for Biomedical Research Projects involving Humans of the Chinese Academy of Medical Sciences & Peking Union Medical College (CAMS&PUMC-IEC-2020–025) on September 14, 2021.

#### Part I

A qualitative research design using the CFIR was adopted to ascertain barriers and facilitators of SLIV. This part was conducted and reported following the Consolidated Criteria for Reporting Qualitative Research (COREQ) guidelines [19].

#### Part II

Using the CFIR-ERIC matching tool, implementation strategies were systematically developed to mitigate barriers and leverage facilitators of SLIV delivery.

### Theoretical framework

#### Part I

The CFIR was used to guide the development of interview guides and thematic analysis [20]. The framework is a meta-theoretical framework that synthesizes constructs from a range of theories about dissemination, innovation, organizational change, implementation, knowledge translation, and research uptake. It has been used to explore barriers and facilitators and assess implementation contexts of various vaccination programs in other contexts [21–23]. CFIR comprises five domains: (1) innovation characteristics (e.g., the evidence for delivering the SLIV program), which was defined as the annual SLIV delivery model in Beijing rather than the vaccine itself; (2) outer setting (e.g., needs and resources of parents for SLIV), which mainly refers to the macro-political environment, governments (e.g., the health care center for primary and secondary school affiliated with district-level Department of Education, and district-level Center for Disease Prevention and Control (CDC), and the Community Health Center), and parents; (3) inner setting (e.g., networks and communications about SLIV, priority, and resources available for implementation in schools), which mainly refers to schools; (4) characteristics of individuals (e.g., knowledge and beliefs about SLIV); and (5) process of implementation (e.g., planning and engaging).

#### Part II

Implementation strategies are methods or techniques used to enhance the implementation of a program or practice. The ERIC is a list of 73 discrete implementation strategies [24, 25]. The CFIR-ERIC Matching tool, developed in 2019 based on expert consensus, is a methodology used to align identified barriers to implementation strategies within CFIR [26, 27]. The tool leads to a structured and evidence-informed selection of strategies to overcome barriers and optimize facilitators, thus improving the implementation process and outcomes in their respective research contexts. The tool has been used to develop implementation strategies for addressing CFIR-based barriers in previous studies [28–30] and is available at https://cfirguide.org/ [27].

### Context and settings

**Fig. 1:**
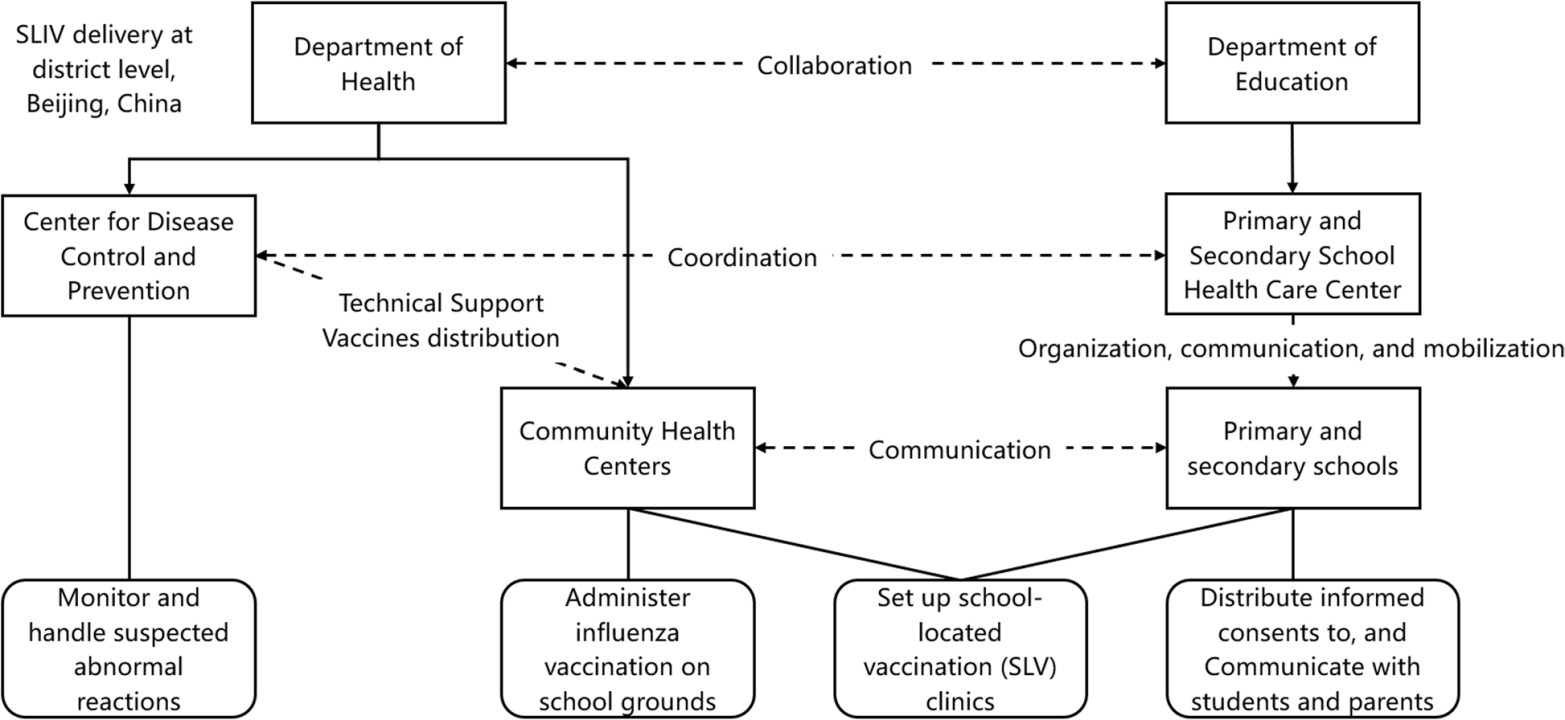
SLIV delivery model at district level in Beijing, China

From September 21 to December 31, 2021, individual semi-structured interviews were conducted with stakeholders from schools to district-level Departments of Education and Health involved in the SLIV program in Beijing, China. The government has implemented the SLIV program in primary and secondary schools in Beijing since 2007, at no expense to individuals and schools since 2009. The program delivery called for a joint effort of the Department of Health and the Department of Education. The Beijing Department of Health was responsible for vaccine procurement and allocation. A detailed delivery model at the district level is displayed in Fig. 1. At the outermost level are the district-level CDC who were responsible for carrying out the SLIV program in collaboration with the Department of Education, distributing vaccines to the community health centers, coordinating community health centers to administer influenza vaccination on school grounds, and monitoring and handling possible suspected abnormal reactions [31]. Community health centers liaised with schools directly to organize dates and set up school-located vaccination clinics, and they were responsible for the actual vaccination of students. Next, the district-level primary and Secondary School Health Care Center or other departments affiliated with the Department of Education at the district level were responsible for the organization, communication, and mobilization of influenza vaccination with schools [31]. At the inner-most level, school physicians were responsible to organize and mobilize staff and students and to guide class headteachers to inform parents and collect consent form for seasonal influenza vaccine.

### Participants and sampling

We included diverse stakeholders who play a vital role in the SLIV program. Purposive sampling and snowball sampling were adopted to recruit interview participants.

#### Government representatives

We included government representatives of both the Department of Health (e.g., district-level CDC, community health center) and the Department of Education (e.g., health care center for primary and secondary school). Government representatives were initially identified according to the institution list of the Work Plan for Influenza Vaccination in Beijing in 2020 [32] document and then decided based on the research team group discussion. To seek the participation of government representatives, the principal investigator of the study (JZ) contacted them directly by telephone.

#### School representatives

School representatives included school physicians, class headteachers, and parents. We defined school as the analysis unit for comparing barriers and facilitators of implementation and included two schools representing low and high performance. Based on the performance in implementing SLIV (defined by vaccination coverage) from 2019 to 2021, one high-performing school with vaccination coverage of 80–90%, ranking in the highest quartile, and one low-performing school with vaccination coverage of 20–50%, ranking in the lowest quartile, were selected. The principal of selected schools recommended their school physician and class headteacher representatives to take interviews. At last, the enrolled school physicians further provided contact information of five to ten parents, including parents of vaccinated students and parents of unvaccinated students in the last influenza season, in each school. School representatives were contacted directly by telephone by student investigators (RY, HW, YH). One parent of a vaccinated student declined to participate due to a time conflict.

### Data collection

The semi-structured interview guides (see Additional file 1) were tailored to different stakeholders and then piloted within the research team and with one school physician, one parent, and one representative from the Department of Education. The pilot helped us to refine the structure of interview guides and clarify some interview questions. Related supporting materials were collected after interviews (e.g., pictures, documents, and consent forms for the seasonal influenza vaccine). All interviews were conducted online in Mandarin Chinese and using Tencent Meeting (Chinese online meeting software, similar to Zoom) or phone because of COVID-19-related physical distancing restrictions. We asked participants to find a quiet place alone to take part in the interview, such as at home or in the workplace where they can share views freely and confidentially without feeling influenced by others. All the participants were informed of the study purpose, the confidentiality of their contributions, the voluntary nature of their participation, and their right to withdraw at any time at the first contact. Oral informed consent was obtained before each interview.

All interviews were carried out by researchers with experience in qualitative research and interviews (XY or XX, both are professors, PhD, female) with at least one note-taker (HW, RY, YH, all are female master’s students). All individuals involved in the study were trained to (1) raise awareness about confidentiality, privacy, and the ethical considerations associated with data collection; (2) avoid pre-assumption of the attitudes of the participants; and (3) hold a neutral stance and not express judgment for the views of the participants. The researchers and the participants did not know each other before the interview. No repeat interviews were conducted. All interviews were recorded and transcribed verbatim. Confidentiality was assured by using roles and numbers instead of names (e.g., school physician 01 and governmental official 02) and removing identifiable information from the transcripts. Data collection ceased after reaching thematic saturation when no further themes were identified [33–36].

### Data analysis

#### Part I: Analyzing the interview transcripts

Data analysis occurred concurrently with data collection. Analysis of early interview transcripts was conducted before or during the recruitment and interviewing of later participants to inform the interview process. The interview transcripts were coded using both inductive and deductive methods in NVivo 12.0 (QSR International, Melbourne, Australia) [37]. First, transcripts were independently read by two team members (HW, RY) with experience in qualitative research to identify preliminary codes (inductive approach). Second, codes with similar meanings were independently clustered to form subthemes and themes and then linked to relevant theoretical constructs in the CFIR (deductive approach) by these same two coders. The coding book was finalized by constant comparison until no new concepts emerged and all conceptual codes were linked to CFIR domains. The CFIR codebook template, which provides codebook descriptions for each construct, guided the development of our study codebook [27]. Third, the two coders independently rated each construct and constantly made comparisons to identify constructs as barriers, facilitators, or neutral factors. Constructs were rated as [1] barriers if they impeded progress or created obstacles, [2] facilitators if they enabled or promoted implementation, and [3] neutral factors if they had no bearing on the implementation. If the comments were mixed, we employed a valence indicator based on the majority of comments to capture the overall sentiment within these constructs. For interpretation, a comparison of ratings across the units of analysis was conducted using a valence matrix. Government participants provided feedback on the findings. Consensus on code names and meanings, valence, and strategies was reached through discussion and review, as well as research team meetings. Data were analyzed in Chinese. All themes, subthemes, codes, and associated illustrative quotations were translated into English for publication purposes.

#### Part II: Application of the CFIR-ERIC tool

After identifying the barriers and facilitators to implementing SLIV, the CFIR-ERIC matching tool, an Excel download online at https://cfirguide.org/ [27], was applied. All CFIR-based barriers were entered, generating an output table with the ERIC strategies and a percentage matched to the CFIR construct. This percentage indicated the strength of endorsement for each proposed strategy associated with barriers identified in part I. For each barrier, strategies color-coded as “green,” indicating ≥ 50% of implementation experts who developed the tool endorsed these strategies to address the barrier, were selected as general recommended strategies. Then, the research team selected and grouped some applicable and practical strategies tailored to Chinese SLIV contexts based on team meetings, stakeholder meetings, and expert consultation.

## Results

For the purpose of this research, information saturation was perceived to be reached after completing interviews with 24 stakeholders, consisting of government representatives (*n* = 3), school physicians (*n* = 2), class headteachers (*n* = 3), and parents (*n* = 16). Of the parents, eight had consented to vaccinate their children, and the other eight declined in the 2021–2022 influenza season. The structure of school participants are summarized in Table 1. The interviews lasted 24 ± 11 min on average.

**Table 1:** Structure of school participants

	**High-performing school (*n*)**	**Low-performing school (*n*)**
School physician	1	1
Class headteacher	2	1
Parent		
Consented vaccination	5	3
Declined vaccination	1	7
**Total**	**9**	**12**

### Part I: Factors across five CFIR domains

Factors influencing the implementation of the SLIV program existed across all five domains of the CFIR. Themes, subthemes, and their valence are summarized in Table 2.

**Table 2:** Subthemes under CFIR constructs by facilitators and barriers, and implementation strategies matched using CFIR-ERIC tool

CFIR domains and constructs		High-performing school	Low-performing school	Implementation strategies^a^
Innovation characteristics	Evidence strength and quality	Implementers emphasized solid evidence on the effectiveness of SLIV ( +)	Implementers recognized evidence on the effectiveness of SLIV ( +)	
Outer setting	Needs and resources of parents	Parents were satisfied with easy access and timely reminders ( +) Generally, parents attached more importance in children’s influenza vaccination during COVID-19 pandemic ( +) Parents perceived susceptibility of influenza (+ *) Parents perceived severity of influenza (+ *) Parents perceived barriers to vaccination, such as misunderstanding about risks of influenza vaccine (− *) Parents perceived benefits of influenza vaccine (+ *)	Parents were satisfied with easy access and timely reminders ( +) Generally, parents attached more importance in children’s influenza vaccination during COVID-19 pandemic ( +) Parents perceived susceptibility of influenza (+ *) Parents perceived severity of influenza (+ *) Parents perceived barriers to vaccination, such as misunderstanding about risks of influenza vaccine (− *) Parents perceived benefits of influenza vaccine (+ *)	Obtain and use parents and family feedback (76%) Involve parents and family members (71%) Conduct local needs assessment (57%)
	Cosmopolitanism	Clear responsibilities and close collaboration among the Department of Health, Department of Education, and schools ( +) Top-down authority ( +) Challenge of arranging time for vaccine administration and influenza vaccine supply ( −)	Clear responsibilities and close collaboration between the Department of Health and Department of Education ( +) Top-down authority ( +) Challenge of arranging time for vaccine administration and influenza vaccine supply ( −)	Build a coalition (62%) Promote network weaving (50%) Develop academic partnerships (50%)
	External policy and incentives	SLIV policy has been in place for many years and was valued by governments ( +)	SLIV policy has been in place for many years and was valued by governments ( +)	
Inner setting	Networks and communications	Efficient communications in school ( +) Parents focused on information related to children, and responded quickly ( +)	Efficient communications in school ( +) Parents focused on information related to children, and responded quickly ( +)	
	Implementation climate			
	Compatibility	School implementers deemed SLIV as routine work ( +)	School implementers deemed SLIV as routine work ( +)	
	Relative priority	School gave priority to SLIV ( +)	School gave priority to SLIV ( +)	
	Readiness for implementation			
	Access to knowledge and information	Training by the Department of Health and Department of Education at the district level to guide school physicians to organize, educate, and mobilize for SLIV was insufficient ( −) School physicians trained teachers, educated students, and provided the class headteachers with targeted materials containing contents and skills to communicate with parents ( +)	Training by the Department of Health and Department of Education at the district level to guide school physicians to organize, educate, and mobilize for SLIV was insufficient ( −) Training and education to teachers and students were undervalued by school physicians ( −)	Conduct educational meetings (79%) Develop educational materials (59%) Distribute educational materials (55%)
Individual characteristics	Knowledge and beliefs about the innovation	School physician’s competence and enthusiasm for implementing SLIV ( +)		
	Individual identification with organization	Parents trusted in the school and government ( +)	Parents trusted in the school and government ( +)	
Process	Planning	School physicians and community health center staff routinely implemented annual SLIV according to notification from the Department of Health and the Department of Education (0) SLIV was not affected because it was scheduled early ( +)	School physicians and community health center staff routinely implemented annual SLIV according to notification from the Department of Health and the Department of Education (0) SLIV was severely delayed due to conflicts with COVID-19 vaccination schedule ( −)	Develop an implementation blueprint (73%) Conduct local needs assessment (50%)

“ + ,” “0,” or “ − ” denotes the theme as a facilitator, neutral factor, or barrier, respectively; a “*” was added if comments were mixed

^a^The percentage indicates the strength of endorsement for each proposed strategy associated with barriers identified

#### Innovation characteristics

The relevant CFIR construct in this domain was *evidence strength and quality*, which facilitated implementation performance in both schools. Participants of the Department of Health and Department of Education, the school physician, and class headteacher in the high-performing school emphasized the effectiveness of SLIV in reducing influenza and school febrile illness outbreaks; participants of the low-performing school expressed similar opinions but at a lesser extent.

The Department of Education in our district has been advocating that if the class vaccination rate was high and reached the herd immunity threshold, febrile outbreaks in schools can be effectively prevented and mitigated. It indeed had an immediate effect (School physician, 01).

#### Outer setting

##### Needs and resources of parents

A majority of participating parents favored the SLIV program for its geographical accessibility and giving priority to vaccinate children early annually. They also stated that schools provided necessary vaccination information, such as the do’s and don’ts before and after vaccination. In addition, they were satisfied with the step-by-step instructions and timely reminders from schools. Overall, the subthemes within this construct facilitated vaccination coverage in schools.

Class headteachers would tell parents to write down their phone number and children’s special condition on the consent form for seasonal influenza vaccine if they were unsure whether their children were eligible for the vaccine. Because on the day of vaccination, the health professionals of the Community Health Center who administered influenza vaccines would call them to verify and decide whether the student was eligible. So, if parents intended to vaccinate their children, they could choose ‘I agree’ on the consent form issued in advance. (School physician, 02)

My child got vaccinated in school this semester. Generally, if we missed it, we might not go to the Community Health Center for vaccination later because time limit. We were busy at work, and my child also needed to go to school on weekdays. (Parent, 01)

Generally, parents attached more importance to children’s influenza vaccination during the COVID-19 pandemic, which was a facilitator. Most of the parents believed that their awareness of influenza and vaccines has improved because of the pandemic; more importantly, influenza vaccination could prevent their children from school absenteeism during this period. However, some parents considered that non-pharmaceutical interventions against COVID-19 could also prevent influenza.

There was a huge increase in demand for influenza vaccines last year because of the COVID-19 pandemic. Influenza vaccines have been in short supply in recent years. (Government representative, 03)

Parents’ perception of susceptibility and severity of influenza, and the benefits and risks of influenza vaccination had a mixed impact on children’s vaccination. First, some parents expressed unawareness of the infectivity of influenza, complacency about children’s health condition, and free rider tendency; others believed that children were susceptible to influenza, that schools were crowded public places where influenza spread easily, that children with poor immunity were more susceptible to influenza, and that non-pharmaceutical interventions had limited effect compared to vaccine. Second, some parents confounded influenza and common cold, and were unaware of influenza-related serious symptoms; others could distinguish influenza from common cold, could understand severe symptoms, and were concerned about absenteeism caused by influenza. Third, some were convinced of the effectiveness of the influenza vaccine; others doubted instead. Fourth, some parents were unsure about their children’s eligibility for vaccines and were concerned about the adverse effects of the vaccine; others expressed their understanding of the safety profile of influenza vaccine and the small probability of adverse events following immunization, as well as their belief in well-established production technology and rigorous approval procedures of influenza vaccine.

##### Cosmopolitanism

This construct facilitated SLIV implementation in several aspects. Participants suggested that the responsibilities of the Department of Education, Department of Health, and schools were clearly defined, and well-connected networks and formal communication channels among them ensured their close collaboration. Participants from the Department of Health and schools stated that community health centers assigned special person to communicate with school physicians of the corresponding schools. In addition, participants from the Department of Education and schools cited that the health care center for primary and secondary school affiliated with the district-level Department of Education issued official documents to schools through an office automation system, and the top-down authority facilitated SLIV implementation. Moreover, the school physician of the high-performing school mentioned that under the unified deployment of the district-level Department of Education, they supervised class headteachers with the support of school leaders and cooperated well with the corresponding community health center. The school physician of the low-performing school, however, stated that the district-level Department of Education rarely interacted with them and that it was mainly the community health center notified the school about SLIV.

We are affiliated with district-level Department of Education. Our duties have much crossover with the district-level CDC, and we need to coordinate with the CDC whenever necessary. (Government representative, 01)

We have an Office Automation System of Department of Education that acts as a formal communication channel between Health Care Center for Primary and Secondary School and schools in our jurisdiction. We issue documents to schools and there is a specialized office or school webmaster in each school to receive the documents and forward them to relevant staff., For example, documents related to health affairs will be sent directly to the school physicians or school principals in charge of health affairs. (Government representative, 01)

However, there was a barrier within this construct: participants mentioned the challenge of arranging school vaccination dates. A common cause for this challenge was an insufficient supply of influenza vaccine, as cited by a government representative from the district-level CDC. In addition, the heavy workload of community health centers and school physicians in September and October, inaccurate forecast of vaccine demand for government procurement based on parent surveys, and short vaccination timeliness made it more difficult to arrange the school vaccination date, according to the representatives from the Department of Health and school physicians.

Last year (2020-2021 influenza season), some schools had not delivered school vaccination until the end of November, when it was already winter and the influenza season had already arrived. This might be attributed to not having a good plan for vaccine supply. It is also true that influenza vaccines have been in short supply sometimes in previous years. (Government representative, 02)

##### External policy and incentives

This construct facilitated implementation performance. Participants acknowledged that the Beijing government emphasized the importance of SLIV for many years. The Chinese CDC has been releasing *Technical Guidelines for Seasonal Influenza Vaccination in China* annually since 2018. The Beijing Department of Health, the Beijing Department of Education, and other relevant departments have been jointly issuing *Work Plan for Influenza Vaccination in Beijing* annually in recent years. The work plan highlighted the importance of SLIV and provided detailed roles and responsibilities of related governmental sectors in implementing SLIV.

Beijing has always attached great importance to influenza vaccination among primary and secondary school students. For over ten years, the Beijing government has provided free influenza vaccination to students in schools. Primary and secondary school students have been included as priority groups for influenza vaccination in Beijing. (Government representative, 02)

#### Inner setting

##### Networks and communications

This construct facilitated SLIV implementation in both schools. According to reports from school physicians, class headteachers, and parents, WeChat (Chinese social communication software similar to WhatsApp and Snapchat) was an efficient communication platform for them. In addition, parents stated that notification from class headteachers would receive quick responses from them since they paid attention to all information related to their children.

As long as the message is related to my children, I will read it. (Parent, 02)

##### Compatibility

The construct was identified as a facilitator at both schools. Participants stated SLIV was their routine work.

It’s part of my job. (Class headteacher, 02)

##### Relative priority

The construct was also a facilitator at both schools. Participants, especially the school physician in the high-performing school, stated that they gave priority to SLIV.

We have many students in our school, so we have always been very strict in the prevention and control of infectious diseases. If influenza occurs in the school, too many students can get infected. So, we must control it at the very beginning. (School physician, 01)

##### Access to knowledge and information

Some participants stated that brief training sessions about SLIV for school physicians and principals in charge of health affairs were conducted by the district-level Department of Education and Department of Health in certain years. Nevertheless, school physicians expressed that the training was specifically for medical professionals and was not targeted to parental concerns. They also noted a lack of detailed guidance for them and class headteachers in organizing the events and mobilizing and communicating with parents.

District-level Department of Education informed us that students needed to get the influenza vaccination, and it’s time for schools to arrange it and communicate with Community Health Centers. They just provided this information, while how to implement it was largely up to each school. (School physician, 01)

In the high-performing school, participants noted that the school physicians trained teachers, educated students, and provided the class headteachers with targeted materials containing contents and skills to communicate with parents. The materials addressed parental concerns by conveying information about the effectiveness and safety of influenza vaccines and information on influenza vaccines used in SLIV (e.g., vaccine manufacturers, vaccination venues). They also mentioned that communications with parents and students should be in line with the SLIV timeline and take advantage of multiple formal channels (e.g., face-to-face parent-teacher meetings, online communication platforms like WeChat, school broadcasts, and health education courses). They considered it was important to communicate with parents 1 to 2 days before filling out the consent forms. In contrast, the school physician in the low-performing school undervalued the importance of providing information. Correspondingly, parents in low-performing schools expressed a lack of publicity and communication about SLIV. This factor acted as a barrier and a facilitator in the two schools.

Every year, we would broadcast in school to educate students about the benefits of influenza vaccination, and motivate teachers to actively engage students and parents. This event was led by us (school physicians), and the broadcast script was written by ourselves. (School physician, 01)

It was not a requirement by the Department of Education. However, based on my work experience, I knew it was time to prepare, so I paid more attention to relevant information and saved useful information. Hence, I could send it to headteachers directly at an appropriate time. (School physician, 01)

School physicians prepared materials about information that parents were concerned about, as well as skills of communication with parents. Then I forwarded this information to parents. (Class headteacher, 01)

#### Individual characteristics

##### Knowledge and beliefs about the innovation

The construct facilitated implementation performance in the high-performing school. The school physician mentioned the facilitation of their rich experience and skills in implementing SLIV. For example, they simplified and optimized the way to collect consent forms for seasonal influenza vaccines according to practical experience.

As far as I know, in some schools, parents were first asked to indicate their willingness to get their children vaccinated in their class WeChat group, and then class headteachers would send the consent forms for the seasonal influenza vaccine to parents willing to vaccinated their children. Instead, we distributed the consent forms to all parents. On the day of distribution, the class headteachers would publicize and mobilize parents, and collect the consent forms the next day. In order to avoid parental conformity behavior, we never collected their willingness online in advance. For example, if one parent said he/she did not want to vaccinate his/her children, some parents may follow, then mobilization of schools would be very passive. (School physician, 01)

Moreover, the school physician in the high-performing school cited that they set goals for influenza vaccination coverage and established statistical accounts to effectively supervise class headteachers to conduct relevant works and provide feedback timely. It was a strong facilitator in improving the influenza vaccination uptake in the school.

We also had a table of our own, like a small statistical account, including phone numbers of class headteachers, the number of students and the expected number of vaccinations in the class, etc... After vaccination, we calculated the coverages for each class, each grade, and the whole school. Our coverage goal for our school was over 85%. (School physician, 01)

##### Individual identification with organization

This construct facilitated implementation in both schools, as most parents expressed confidence in the SLIV system, the school, and the government.

Since the vaccine was recommended by the government, I thought my child should get it. Winter was coming, accompanied by the influenza season. The program is a government welfare for children, and was arranged collectively by schools. I thought it was great, so I signed the consent form for my child. I want her to get vaccinated every year. (Parent, 03)

#### Process

##### Planning

School physicians and community health center staff noted that they routinely implemented annual SLIV according to notification from the Department of Health and the Department of Education, which was a neutral factor.

Generally, it begins in late September or October. It is usually arranged by the district-level CDC, who notifies us of the start date of vaccine application this year. There is a physician responsible for applies for vaccines in our center. The physician enters the number of vaccines that need to apply into the system, and the delivery company delivers them to us. After the preparation is finished, the physicians responsible for providing vaccination services will schedule a meeting with the school physicians to schedule the dates for vaccine administration, count the number of school vaccinations, and determine the number of physicians and days needed based on the workload. (Government representative, 03)

Since this is a regular work every year, we are usually ready in September and October, but we still need to wait for notification from the Community Health Center before collecting parents’ willingness to vaccinate their children. Basically, we follow the steps of the Community Health Center. For example, they inform us of the approximate date of vaccination in our school, and send us the consent forms in advance. According to the scheduled time, we generally send the consent forms to parents four or five days in advance, collect the forms the next day, and preliminarily check the forms as vaccination approaching. (School physician, 02)

Planning influenced SLIV implementation when SLIV coincided with the COVID-19 vaccination in schools in Beijing in late October and November 2021. Participants consistently expressed a higher priority for COVID-19 vaccination over influenza vaccination. They stressed that the implementation performance of SLIV varied widely among schools in the 2021–2022 influenza season. In the high-performing school, participants stated that the SLIV was scheduled early by the district-level Department of Education and Department of Health to allow ample time for COVID-19 vaccination. Thus, the SLIV was not affected and completed as expected. However, in the low-performing school, participants stated that the schedule of SLIV conflicted with COVID-19 vaccination, which resulted in the delay of SLIV until December when Beijing entered the epidemic period for seasonal influenza and other respiratory infectious diseases. The school physician at the low-performing school considered it was the primary reason for low influenza vaccination coverage.

It was arranged early in our district, so it was not affected; otherwise, it would be very difficult and more stressful. (School physician, 01)

Our school’s influenza vaccination rate was low this year, mainly because it conflicted with the COVID-19 vaccination. District-level Department of Health and Department of Education worked to ensure only students eligible for vaccination can get vaccinated. You couldn’t get a flu shot until 14 days after the second dose of the COVID-19 vaccine. (School physician, 02)

When the school started vaccination against COVID-19, my child caught a cold. Two weeks later, when he recovered, he got the first shot of the COVID-19 vaccine and the second shot subsequently. When the school told us to get the flu shot, it was already close to the end of semester, so I decided not to get him vaccinated this year. (Parent, 04)

### Part II: Implementation strategies

The results of the CFIR-ERIC matching tool are provided in the last column of Table 2 (more details in Additional file 2). Given that strategies based on the cumulative percent for all barriers may not be specific, the implementation strategies were chosen based on the percent for each specific barrier. For the barrier existing in *patient needs and resources* (at the consumer level), recommended strategies included “involving parents and family members,” “obtaining and using parents and family feedback,” and “conducting local needs assessment.” For the barrier of lack of *cosmopolitanism* (at the system level), recommended strategies included “building a coalition,” “developing academic partnerships,” and “promoting network weaving.” For the barrier of lack of *access to knowledge and information* (at the school level), recommended strategies included “conducting educational meetings,” “developing educational materials,” and “distributing educational materials” which belonged to the ERIC cluster of “training and educating stakeholders.” For the barrier of lack of *planning* (at the system level), recommended strategies included “developing a formal implementation blueprint” and “conducting local needs assessment.”

After consideration of SLIV contexts in Beijing through research team meetings, key stakeholder meetings, and expert consultation, we tailored applicable and practical strategies at the system, school, and consumer levels. At the system level, in the face of a lack of *planning and cosmopolitanism*, the underlying implementation strategies of “developing an implementation blueprint” and “promoting network weaving” may help the early and overall planning process and facilitate information sharing and collaboration on issues such as vaccine supply and dates for vaccine administration to achieve a shared goal of SLIV implementation within and outside the Department of Health and Education, schools, and vaccine companies. Other strategies like “building a coalition,” “developing academic partnerships,” and “conducting local needs assessment” may be less targeted and coalitions have already existed. At the school level, training and education school implementers through educational meetings and providing materials may be needed. At the consumer level, engaging students and parents in SLIV implementation to address parental needs may be of vital importance.

## Discussion

To our knowledge, this study is the first to explore the barriers and facilitators in implementing SLIV in Chinese contexts drawing on the perspectives of multiple stakeholders. Facilitators identified by stakeholders existed across all five domains of CFIR and included solid evidence-base, easy access, needs promoted by COVID-19, clear responsibilities and close collaboration among the Department of Health and Department of Education, top-down authority, integration of SLIV into the routine of schools, the relative priority of SLIV, efficient communication within schools, and parents’ trust in school and government. On the other hand, barriers were mainly related to the CFIR domains of outer setting (i.e., needs and resources of parents, and cosmopolitanism), inner setting (i.e., access to knowledge and information about the SLIV), and process (i.e., planning). Understanding the implementation barriers from a theoretical perspective is important to identify solutions to overcome them. This study proposed context-specific implementation strategies that may be suitable for systematically addressing barriers to the SLIV delivery in the free SLIV program in Beijing based on CFIR-ERIC.

### Plan and coordinate at the system level

Our findings indicated that a lack of integrated plan and coordination was likely to hinder SLIV. First, the plan and coordination process was limited by vaccination supply, which was a common barrier as suggested by previous studies [18], especially in low- and middle-income counties and during COVID-19 [38]. Second, school physicians faced challenges to arrange proper school vaccination dates, which was consistent with previous studies [39–41]. They needed to consider the tight timelines to protect children before the influenza season and balance other school activities like exams and other vaccination plans [42]. Third, the COVID-19 pandemic, although generally increased influenza vaccination rates, magnified the problem of ineffective planning and coordination [38]. A study conducted in Hong Kong also suggested that the school staff were stressed about school vaccination during the COVID-19 pandemic [42].

The corresponding implementation strategy suggested by ERIC and tailored to the contexts were “developing an implementation blueprint” and “promoting network weaving.” It is important to take advantage of the existing high-quality working relationships and networks among multi-stakeholders (e.g., Department of Education, Department of Health, schools, Community Health Centers, vaccine manufacturers), so as to promote information sharing and schedule school vaccination dates smoothly. Similarly, a study in the USA revealed that successful SLIV programs required adequate and advanced plan and coordination, even potentially through a dedicated program coordinator in the school or district [43]. In addition, it is important to provide supportive supervision with quick feedback and implementation tools in supporting broad-scale quality improvement programs [44].

### Train and educate stakeholders at the school level

We found that school physicians’ capacity, skill, and experience were crucial facilitators for SLIV. This was consistent with the findings from a systematic review of school-based vaccination programs in high-income countries that found school physicians’ roles and experiences were common factors influencing the implementation of programs [18]. School implementers are influential local “connectors” for SLIV because they know specific students and parents and have access to educate families about SLIV [41]. The present study indicated that some school implementers lacked the capacity to communicate with families about the influenza vaccine and that training by the district-level Department of Health and Department of Education to support them was limited. This was similar to the barrier in a previous study in Hong Kong, revealing that due to inadequate medical knowledge, school implementers were generally passive in communicating with parents and students [42].

Implementation strategies under the cluster of “training and educating stakeholders” (i.e., conducting educational meetings and developing and distributing educational materials) were suggested by ERIC to be of relatively high importance and feasibility [35]. These potential strategies providing education to school implementers might be useful. Education should focus on increasing school implementers’ knowledge, skills, and confidence in communicating with parents and students [42]; educational materials could be manuals, toolkits, and other supporting materials. Education should detail how to organize, educate, and mobilize staff and families to keep the workload to a minimum [45]. The SLIV practice in the United Kingdom (UK) [39] is worth learning from government and relevant institutions developed education packs to provide useful information on influenza [46, 47] and communication packs to help better communicate and engage with schools and families [46, 48].

### Engage students and parents at the consumer level

Evidence indicated that communicating with parents about their concerns with vaccination and obtaining parental consent were crucial for the success of the SLIV program [18]. Our study found that influenza vaccination of children may be affected by parents’ perceived susceptibility and severity of influenza, as well as perceived benefits and safety of influenza vaccine. Similarly, previous studies suggested that parents’ lack of awareness about the threat of influenza was a common barrier [18] and that their primary concerns were centered on influenza vaccine adverse events [18, 43, 45]. However, our study reaffirms that parents do not have adequate knowledge related to influenza to help them make a decision to get their children vaccinated; efforts should be made to increase parents’ awareness of the importance of influenza and its vaccine in order to fully implement the SLIV and increase the uptake of influenza vaccination.

Parental needs could be addressed by potential implementation strategies of “conducting local needs assessment,” “involving parents and family members,” and “obtaining and use parents and family feedback” to engage them in SLIV activities, which were of relative high importance and feasibility suggested by the ERIC study [25]. Indeed, schools need to conduct educational activities in an attractive and easily understood way according to the SLIV timeline [43, 45]. For example, the UK government provided targeted materials and teaching toolkits, such as stickers, banners, videos, and electronic communication materials to ensure the sessions were interesting and informative [39]. Meanwhile, it is also necessary to pay attention to the perspectives of students and parents [18], such as to understand reasons that parents hesitate to vaccinate their children and develop materials based on their needs (e.g., side effects of influenza vaccination). Behavior change theory in school vaccination programs could also be used [18].

### Strengths and limitations

This study may be the first to identify barriers and facilitators to implementing SLIV in China. Multiple stakeholders were interviewed, and related policy documents were collected, allowing us to triangulate findings and capture divergent perspectives. The CFIR provided the research team a systematic process to follow, a consistent template which allowed for multiple coders to be involved in analysis, and a common language across study components. The findings provide valuable insights into the delivery of school-located vaccination programs and policies. Further research is needed to verify the abovementioned implementation strategies of SLIV in real-world settings.

This study has several limitations. First, there is a limitation of the generalizability of the results of qualitative research. However, qualitative research aims to capture rich and in-depth information and improve understanding of the phenomenon of interest. Second, given that interviews were audio recorded, social desirability bias was possible, and views or perceptions from participants may not totally reflect the real implementation process. We addressed this by presenting participants with information detailing the anonymity of the data and ensuring that the interviewers were well-trained and signed confidentiality agreements before interviewing. Third, transcripts were not returned to participants for comments or corrections. Fourth, because of the COVID-19 pandemic, all interviews were conducted online, which hindered observations of participants’ non-verbal language during the interviews. Finally, implementation strategies recommended by the CFIR-ERIC matching tool were based on expert consensus, and mechanisms of effectiveness may be vague. We tried to involve multiple stakeholders in the study and tailored the general strategies to practical strategies through meetings with team members, key stakeholders, and experts.

## Conclusions

Overall, based on stakeholder’s perspectives and experiences, the SLIV program appeared to have been implemented smoothly in the current context, while some barriers have been identified. Limited training to support school implementers, parents’ misconception, inefficient coordination for vaccine supply and vaccination dates, and inadequate planning adversely impacted SLIV. These factors may have led to low and differential coverages in SLIV performance among schools. To improve the quality of SLIV and promote the uptake of vaccination coverage, planning and coordinating at the system level, training and educating school implementers at the school level, and engaging students and parents to address parental concerns at the consumer level may be important.

### Supplementary Information

**Additional file 1. **Interview guides.

**Additional file 2. **Prioritized list of strategies generated by the CFIR-ERIC Matching tool.

## Data Availability

All the data and materials of this qualitative study are available from the corresponding author upon reasonable request.

## Abbreviations

SLIV: School-located influenza vaccination
CFIR: Consolidated Framework for Implementation Research
ERIC: Expert Recommendations for Implementing Change
CDC: Center for Disease Prevention and Control
UK: United Kingdom
