# Supplementary material for: Identifying implementation strategies to address barriers of implementing a school-located influenza vaccination program in Beijing
Source: Implement Sci Commun. 2023 Oct 11;4:123. doi: 10.1186/s43058-023-00501-8 (PMC10566160; doi:10.1186/s43058-023-00501-8)
Supplement: Supplementary file 1 — Additional file 1. Interview guides. [file 43058_2023_501_MOESM1_ESM.docx]

# Additional file 1 -- Interview guides

Table A1 Interview guide-Education and health sectors at district level (Slightly adapted to interview school physicians and class headteachers)

| Introduction  2 min | Dear Madam/Sir:  Hello! We are the research team members of the School of Population Medicine and Public Health, Chinese Academy of Medical Sciences & Peking Union Medical College. We are studying on "School-Located Influenza Vaccination" program. In this interview, we mainly want to know your views on school-located influenza vaccination and understand barriers and facilitators to improve the program. All the information of this interview will only be used for scientific research, and will never be leaked to anyone else. Please look at the informed consent form for this interview. Do you agree to be recorded? Thank you very much for your support! Before we start the interview, do you have any questions? | | |
| --- | --- | --- | --- |
|  | **CFIR domains** | **Question** | **Make a detailed inquiry** |
| 2 min | Individual characteristics, and innovation characteristics | How long have you been working on school-located influenza vaccination? |  |
|  |  | How do you think about the school-located influenza vaccination? |  |
| 2 min | Outer setting, and inner setting | What was the influenza vaccine coverage for primary school students in recent three years? |  |
|  |  | How much emphasis did the municipal and district government (or your school) attach to the influenza vaccination for elementary school students? | Were there any incentives or goals for districts (or your school) to increase influenza vaccine uptake? |
| 6 min | Process | How did the municipal and district government (or your school) implement influenza vaccination program for elementary school students? | Before, during, after vaccination  Organization, education, mobilization  Please try to describe in details (Who? To whom? When? What? Where? How many times? What data was collected?) according to the timeline, events, and related documents. |
| 4 min | Outer setting, and inner setting | What roles did the Municipal/District Education/Health departments (or different school staff) play in the vaccination for elementary school students? |  |
|  |  | School-located influenza vaccination program involves many families, schools, manufacturers, institutions, and government sectors, can you describe the networks and communication mechanisms among them? |  |
| 4 min | Outer setting, and inner setting | Were schools trained and supervised by district education and health departments?  Was there an education activity about influenza vaccines for primary school students in your district (or your school)?  How did the schools communicate with parents? | Who? To whom? When? What? Where? How? How many times? |
| 1 min | Outer setting, and inner setting | Was the school-located influenza vaccination compatible with your routine work? |  |
| 4 min | Process | How was this year's vaccination process different from previous years? | Vaccination time, vaccine supply?  Why? COVID-19? |
|  |  | How was your work about school influenza vaccination different from previous years? | Why? COVID-19? |
| 2 min | Process | What are the difficulties in the process of influenza vaccination for primary school students? | Were there any stories/examples to share? |
|  |  | What can be further improved? | What approaches can be taken? |
| Concluding  Remarks  3 min | A brief summary, confirm with the respondents | | Is there anything I haven't understand correctly? |
|  | This concludes today's interview. Do you have any other question? Thank you again! | |  |

Table A2 Interview guide-Community health centers

| Introduction  2 min | Dear Madam/Sir:  Hello! We are the research team members of the School of Population Medicine and Public Health, Chinese Academy of Medical Sciences & Peking Union Medical College. We are studying on "School-Located Influenza Vaccination" program. In this interview, we mainly want to know your views on school-located influenza vaccination and understand barriers and facilitators to improve the program. All the information of this interview will only be used for scientific research, and will never be leaked to anyone else. Please look at the informed consent form for this interview. Do you agree to be recorded? Thank you very much for your support! Before we start the interview, do you have any questions? | | |
| --- | --- | --- | --- |
|  | **CFIR domains** | **Question** | **Make a detailed inquiry** |
| 2 min | Individual characteristics | How long have you been working in a community health center? |  |
| 3 min | Outer setting | What are your daily works? | Work load |
|  |  | How many schools are you responsible for influenza vaccinations? | How many children are in these schools? |
| 2 min | Process | Did you set any goal for school-located influenza vaccination coverage? |  |
| 9 min | Process and outer setting | How did the community health centers cooperate with schools about school-located influenza vaccination? | Who is responsible for communicating with schools about school-located influenza vaccination?  When do you go to school to vaccinate elementary school students every year?  What should be attached importance in this process?  What information do you need to collect from schools about school-located influenza vaccination before, during, and after vaccination? |
| 7 min | Process | How many of you went to school at a time for school-located influenza vaccination? |  |
|  |  | Can you talk about the influenza vaccination process at school? | How were the responsibilities assigned among related personnel?  What were your responsibilities? |
|  |  | What were the impressive things during the process? |  |
|  |  | Have you ever encountered any emergencies? | How did you address it/them? |
| 3 min | Process, and outer setting | Are there any differences about school-located influenza vaccination before and during COVID-19 pandemic? | Anything special? |
| Concluding  Remarks  2 min | A brief summary, confirm with the respondents | | Is there anything I haven't understand correctly? |
|  | This concludes today's interview. Do you have any other question? Thank you again! | |  |

Table A3 Interview guide-Parents

| Introduction  2 min | Dear Madam/Sir:  Hello! We are the research team members of the School of Population Medicine and Public Health, Chinese Academy of Medical Sciences & Peking Union Medical College. We are studying on "School-Located Influenza Vaccination" program. In this interview, we mainly want to know your views on school-located influenza vaccination and understand barriers and facilitators to improve the program. All the information of this interview will only be used for scientific research, and will never be leaked to anyone else. Please look at the informed consent form for this interview. Do you agree to be recorded? Thank you very much for your support! Before we start the interview, do you have any questions? | | |
| --- | --- | --- | --- |
|  | **CFIR domains** | **Question** | **Make a detailed inquiry** |
| 2 min | Individual characteristics | Can you talk a little bit about your family, your child's school, class, and did your child got influenza vaccine? |  |
| 6 min | Outer setting | What did you considered when you decided to let your child get/not get the flu shot? |  |
|  |  | Did you try to get any information about flu or flu vaccines before vaccination? | Why? Under what circumstances? |
|  |  | What kinds of information did you focus on? | e.g., incidence and severity of influenza among children; safety, efficacy, and benefits of influenza vaccination; time and place for vaccination; the do’s and don’ts before and after vaccination; vaccine manufacturers, health service institutions and professionals who administer influenza vaccine; vaccination policy; imported or domestic vaccines, trivalent or quadrivalent vaccines; impact of COVID-19; attitudes of other parents |
| 12 min | Process | Can you tell me the process for students to get a flu shot in your child’s school this year? | What's your comment on school-located influenza vaccination? |
|  | Outer setting, and inner setting | What information did you obtain from school about school-located influenza vaccination? | How did the class headteacher communicate with parents? |
|  | Individual characteristics | Do you trust in the information about vaccines provided by the class headteacher? |  |
| 5 min | Process, and outer setting | Was your children’s influenza vaccination influenced by COVID-19 pandemic? |  |
|  | Outer setting | Have your children got COVID-19 vaccination this year? | Why or why not?  Do you have any concerns? |
| Concluding  Remarks  3 min | A brief summary, confirm with the respondents | | Is there anything I haven't understand correctly? |
|  | This concludes today's interview. Do you have any other question? Thank you again! | |  |
