# Supplementary material for: Identifying implementation strategies to address barriers of implementing a school-located influenza vaccination program in Beijing
Source: Implement Sci Commun. 2023 Oct 11;4:123. doi: 10.1186/s43058-023-00501-8 (PMC10566160; doi:10.1186/s43058-023-00501-8)
Supplement: Supplementary file 2 — Additional file 2. Prioritized list of strategies generated by the CFIR-ERIC Matching tool. [file 43058_2023_501_MOESM2_ESM.docx]

# Additional file 2 -- Prioritized list of strategies generated by the CFIR-ERIC Matching tool

Table A4 Prioritized list of strategies generated by the CFIR-ERIC Matching tool

| ERIC Strategies | Cumulative Percent | Patient Needs & Resources | Cosmopolitanism | Access to knowledge & information | Planning |
| --- | --- | --- | --- | --- | --- |
| Conduct local needs assessment | **122%** | **57%** | 12% | 3% | **50%** |
| Conduct educational meetings | **108%** | 10% | 12% | **79%** | 8% |
| Assess for readiness and identify barriers and facilitators | **98%** | 33% | 15% | 7% | 42% |
| Develop a formal implementation blueprint | **95%** | 5% | 4% | 14% | **73%** |
| Create a learning collaborative | **83%** | 0% | 31% | 45% | 8% |
| Build a coalition | **83%** | 14% | **62%** | 3% | 4% |
| Involve patients/consumers and family members | **83%** | **71%** | 4% | 3% | 4% |
| Obtain and use patients/consumers and family feedback | **80%** | **76%** | 0% | 0% | 4% |
| Develop educational materials | **80%** | 10% | 4% | **59%** | 8% |
| Capture and share local knowledge | **79%** | 10% | 23% | 31% | 15% |
| Use advisory boards and workgroups | **79%** | 29% | 35% | 0% | 15% |
| Conduct local consensus discussions | **77%** | 29% | 15% | 10% | 23% |
| Identify and prepare champions | **75%** | 5% | 15% | 24% | 31% |
| Develop academic partnerships | **73%** | 5% | **50%** | 10% | 8% |
| Promote network weaving | **64%** | 0% | **50%** | 10% | 4% |
| Distribute educational materials | **64%** | 5% | 0% | **55%** | 4% |
| Conduct educational outreach visits | **63%** | 5% | 23% | 28% | 8% |
| Conduct ongoing training | **61%** | 0% | 0% | 38% | 23% |
| Visit other sites | **60%** | 0% | 38% | 14% | 8% |
| Provide local technical assistance | 48% | 5% | 4% | 24% | 15% |
| Prepare patients/consumers to be active participants | 48% | 48% | 0% | 0% | 0% |
| Develop and implement tools for quality monitoring | 45% | 14% | 0% | 0% | 31% |
| Facilitation | 45% | 0% | 12% | 10% | 23% |
| Use an implementation adviser | 42% | 5% | 8% | 14% | 15% |
| Develop resource sharing agreements | 38% | 0% | 31% | 3% | 4% |
| Recruit, designate and train for leadership | 30% | 0% | 15% | 3% | 12% |
| Organize clinician implementation team meetings | 29% | 0% | 0% | 14% | 15% |
| Involve executive boards | 28% | 5% | 23% | 0% | 0% |
| Intervene with patients/consumers to enhance uptake & adherence | 28% | 24% | 0% | 0% | 4% |
| Facilitate relay of clinical data to providers | 28% | 10% | 0% | 10% | 8% |
| Work with educational institutions | 26% | 0% | 19% | 7% | 0% |
| Provide ongoing consultation | 26% | 5% | 0% | 17% | 4% |
| Tailor strategies | 26% | 14% | 0% | 0% | 12% |
| Identify early adopters | 26% | 0% | 4% | 10% | 12% |
| Shadow other experts | 25% | 0% | 4% | 21% | 0% |
| Conduct cyclical small tests of change | 25% | 10% | 0% | 3% | 12% |
| Purposely reexamine the implementation | 24% | 5% | 4% | 0% | 15% |
| Obtain formal commitments | 23% | 0% | 19% | 0% | 4% |
| Inform local opinion leaders | 22% | 0% | 15% | 7% | 0% |
| Provide clinical supervision | 22% | 5% | 0% | 17% | 0% |
| Promote adaptability | 21% | 14% | 0% | 7% | 0% |
| Alter incentive/allowance structures | 21% | 10% | 0% | 0% | 12% |
| Centralize technical assistance | 19% | 0% | 4% | 3% | 12% |
| Stage implementation scale up | 19% | 0% | 0% | 3% | 15% |
| Develop an implementation glossary | 18% | 0% | 4% | 7% | 8% |
| Model and simulate change | 18% | 0% | 8% | 7% | 4% |
| Use train the trainer strategies | 18% | 0% | 8% | 10% | 0% |
| Develop and organize quality monitoring systems | 15% | 0% | 0% | 0% | 15% |
| Start a dissemination organization | 12% | 5% | 8% | 0% | 0% |
| Access new funding | 12% | 0% | 4% | 0% | 8% |
| Use mass media | 11% | 0% | 8% | 3% | 0% |
| Make training dynamic | 10% | 0% | 0% | 10% | 0% |
| Create new clinical teams | 10% | 10% | 0% | 0% | 0% |
| Increase demand | 10% | 10% | 0% | 0% | 0% |
| Change record system | 9% | 5% | 4% | 0% | 0% |
| Audit and provide feedback | 8% | 5% | 0% | 3% | 0% |
| Change accreditation or membership reqs | 8% | 0% | 8% | 0% | 0% |
| Create or change credentialing and/or licensure standards | 8% | 0% | 4% | 0% | 4% |
| Use data experts | 8% | 0% | 4% | 0% | 4% |
| Remind clinicians | 7% | 0% | 0% | 3% | 4% |
| Use data warehousing techniques | 5% | 5% | 0% | 0% | 0% |
| Fund and contract for clinical innovation | 3% | 0% | 0% | 3% | 0% |
| Revise professional roles | 3% | 0% | 0% | 3% | 0% |
| Alter patient/consumer fees | 0% | 0% | 0% | 0% | 0% |
| Change liability laws | 0% | 0% | 0% | 0% | 0% |
| Change physical structure and equipment | 0% | 0% | 0% | 0% | 0% |
| Change service sites | 0% | 0% | 0% | 0% | 0% |
| Develop disincentives | 0% | 0% | 0% | 0% | 0% |
| Make billing easier | 0% | 0% | 0% | 0% | 0% |
| Mandate change | 0% | 0% | 0% | 0% | 0% |
| Place innovation on fee for service lists/formularies | 0% | 0% | 0% | 0% | 0% |
| Use capitated payments | 0% | 0% | 0% | 0% | 0% |
| Use other payment schemes | 0% | 0% | 0% | 0% | 0% |
